# Genotype-by-Environment Interactions in Nonalcoholic Fatty Liver Disease and Chronic Illness among Mexican Americans: The Role of Acculturation Stress

**DOI:** 10.3390/genes15081006

**Published:** 2024-08-01

**Authors:** Eron G. Manusov, Vincent P. Diego, Marcio Almeida, David Ortiz, Joanne E. Curran, Jacob Galan, Ana C. Leandro, Sandra Laston, John Blangero, Sarah Williams-Blangero

**Affiliations:** 1 Department of Human Genetics, University of Texas Rio Grande Valley, Brownsville, TX 78520, USAjoanne.curran@utrgv.edu (J.E.C.);; 2 South Texas Diabetes and Obesity Institute, School of Medicine, University of Texas Rio Grande Valley, Brownsville, TX 78520, USA; 3 School of Medicine, University of Texas Rio Grande Valley, Edinburg, TX 78539, USA; david.ortiz04@utrgv.edu

**Keywords:** G×E, NAFLD, Mexican Americans, acculturation stress, marginalization

## Abstract

This study examines the complex interplay of genetic and environmental interactions that shape chronic illness risk. Evidence is mounting for the role of genetic expression and the immune response in the pathogenesis of chronic disease. In the Rio Grande Valley of south Texas, where 90% of the population is Mexican American, chronic illnesses (including obesity, diabetes, nonalcoholic liver disease, and depression) are reaching epidemic proportions. This study leverages an ongoing family study of the genetic determinants of risk for obesity, diabetes, hypertension, hyperlipidemia, and depression in a Mexican American population. Data collected included blood pressure, BMI, hepatic transaminases, HbA1c, depression (BDI-II), acculturation/marginalization (ARSMA-II), and liver health as assessed by elastography. Heritability and genotype-by-environment (G×E) interactions were analyzed, focusing on the marginalization/separation measure of the ARSMA-II. Significant heritabilities were found for traits such as HbA1c (h^2^ = 0.52), marginalization (h^2^ = 0.30), AST (h^2^ = 0.25), ALT (h^2^ = 0.41), and BMI (h^2^ = 0.55). Genotype-by-environment interactions were significant for HbA1c, AST/ALT ratio, BDI-II, and CAP, indicating that genetic factors interact with marginalization to influence these traits. This study found that acculturation stress exacerbates the genetic response to chronic illness. These findings underscore the importance of considering G×E interactions in understanding disease susceptibility and may inform targeted interventions for at-risk populations. Further research is warranted to elucidate the underlying molecular pathways and replicate these findings in diverse populations.

## 1. Background

Hispanics are one of the largest and fastest-growing minority groups in the United States and are burdened with a disproportionately high prevalence of chronic illnesses [1]. In regions such as the Rio Grande Valley of south Texas, the prevalence of nonalcoholic fatty liver disease (NAFLD) approaches epidemic proportions (60%) in the Mexican American population [2,3,4,5]. NAFLD is the leading cause of liver disease in the world [6,7,8,9,10], and risk for the condition is associated with diabetes [11,12,13], depression [14,15], and obesity [8]. The heritability of NAFLD [16] ranges from 25% to 75%, with genetic loci related to hepatic fat accumulation, lipid metabolism, and insulin resistance [17]. Obesity [18], depression [19,20,21,22], and diabetes are also highly heritable [23,24] and influence the prevalence of NAFLD, as well as the progression of liver disease.

Previous research on NAFLD identified G×E interaction effects with depression, socioeconomic status, physical exercise, social stress, and the social determinants of health [25,26,27,28]. As communities on the US–Mexico border grow and change in size, acculturation stress affects individuals as they assimilate into the US population. South Texas is unique compared to many areas of the United States because of its proximity to the US–Mexico border, high rates of poverty, increased immigration, and rapid growth. However, it shares similarities with other areas that experience high migration rates [29]. The population of the Rio Grande Valley region is predominantly of Mexican ancestry and is home to families that have been established for generations, new Mexican immigrants, families that only speak Spanish, bilingual families, and families that only speak English. The counties of the Rio Grande Valley (Cameron, Hidalgo, Starr, and Willacy) rank among the poorest in the nation. Cultural integration in the region is influenced by English language fluency, occupation, income status, and health insurance coverage [30].

The acculturation phenomenon occurs when individuals from one culture come into continuous first-hand contact with a predominant culture, subsequently changing cultural beliefs, values, identity, language, customs, diet, or social relationships [31,32]. The acculturation process, specifically acculturation stress, may result in depression, substance abuse, and less healthy dietary behaviors. Although there are over one hundred models of acculturation, a prominent accepted model is the “four-fold model” that assesses integration, assimilation, marginalization, and separation [33]. This model attempts to explain acculturation in terms of goodness-of-fit into society that can be mediated by acculturation stress and is described in the development of the Acculturation Rating Scale for Mexican Americans-II (ARSMA-II) [34,35]. The ARSMA-II contains two scales, one that measures acculturation as orientation (Mexican or Anglo) (13 items with a coefficient alpha of 0.83) and a second scale that measures marginality and separation (17 items with a coefficient alpha of 0.88). Each scale can be used independently. 

Our goal is to assess the gene-by-environment interaction effects of acculturation stress on chronic illness, obesity, depression, diabetes, and NAFLD in Mexican Americans of the Rio Grande Valley. We theorize that genes underlying chronic illness interact with marginalization (acculturation stress) to worsen disease. 

## 2. Materials and Methods

The University of Texas Rio Grande Valley IRB approved the study protocol. All participants provided informed consent before participating in the study. The Rio Grande Valley (RGV) is 90% Hispanic, with a high prevalence of diabetes (32%), obesity/overweight (60%), and NAFLD (60%). As described in earlier publications, our ongoing genetic epidemiological study recruits probands who are 18 years of age or older, have a family history of diabetes, and have five family members each having four Mexican-origin grandparents who are eligible to participate [3].

We recruited 547 participants into the RGV Family Study belonging to large families [16]. Community health workers (research assistants) interfaced with local community sites and contacted the family members identified by the proband. They obtained informed consent and scheduled each participant for assessment by the investigators at a community health clinic [36]. Information gathered included blood pressure, body mass index (BMI), liver transaminases (AST, ALT), HbA1c, an assessment of depression (BDI-II), a measure of acculturation (The Acculturation Rating Scale for Mexican Americans-II (ARSMA-II), and a measure of liver health (vibration-controlled transient elastography and controlled attenuation parameter measured by the FibroScan device) [37,38]. The recruitment strategy is further described in previous publications [16,25].

The Beck Depression Inventory-II (BDI-II) was used to assess the degree of depressive symptoms over the past two weeks [16,39]. The BDI-II is a reliable screening tool for assessing the severity of depression. The BDI-II assesses the severity of depression and is an acceptable screening instrument for depression when administered either in Spanish or English [40,41,42].

The Acculturation Rating Scale for Mexican Americans-II (ARSMA-II) was designed to quantify the acculturation construct and is the basis of many available acculturation scales. The scale comprises two subscales (orientation and marginalization) that can be analyzed individually. Since we are interested in the role of acculturation stress and G×E effects on NAFLD, we analyzed the marginalization/separation measure of the ARSMA-II.

We measured hepatic fibrosis, which was reported as the Liver Stiffness Measurement LSM Youden Index (kPa), steatosis (controlled attenuation parameter dB/m CAP), and the FAST (FibroScan-AST) (the most predictive model for risk of fibrosis that combines LSM, CAP, and AST). CAP identifies steatosis with an AUROC of 0.87 (95% confidence interval [CI] 0.82–0.92) for S ≥ S1, 0.77 (95% CI 0.71–0.82) for S ≥ S2, and 0.70 (95% CI 0.64–0.75) for S = S3. Youden cutoff values for S ≥ S1, S ≥ S2, and S ≥ S3 were 302 dB/m, 331 dB/m, and 337 dB/m, respectively. LSM identified patients with fibrosis with AUROCs of 0.77 (95% CI 0.72–0.82) for F ≥ F2, 0.80 (95% CI 0.75–0.84) for F ≥ F3, and 0.89 (95% CI 0.84–0.93) for F = F4. Youden cutoff values for F ≥ F2, F ≥ F3, and F = F4 were 8.2 kPa, 9.7 kPa, and 13.6 kPa, respectively (Echosens, Paris, France) [37,43,44,45]. Exclusion criteria included pregnancy, implant, or a cardiac pacemaker. Participants presented fasting for at least 3 h prior to the exam. The participants lay supine, face-up on the exam table, and fully abducted their right arm. The FibroScan automatically chooses the correct probe size (M/XL). Ultrasound conduction gel was applied to the abdomen at the 8th–10th intercostal rib space at the mid-axillary line. Measurements were performed by scanning the right liver lobe through the intercostal space. CAP is an average estimate of ultrasound attenuation at 3–5 MHz (dB/m). Liver stiffness measurements (LSMs) are an average stiffness measurement at a shear wave frequency of 50 Hz (kilopascals). The median value of successful measurements with at least ten validated measurements and a success rate of at least 30% were selected to represent the LSM [44,46,47,48].

Additional measures included body mass index (BMI), aspartate aminotransferase (AST) (AST > 37), alanine aminotransferase (ALT) (ALT > 40 U/L in males or AST or ALT > 31 U/L in females), and the AST/ALT ratio (AST/ALT ratio > 1) [49]. The FibroScan-AST score (FAST), to evaluate progression of liver disease, was calculated for each participant [44].

### 2.1. Statistical Analysis

Using a variance components approach, we estimated heritabilities (h^2^) and genotype x environment interaction effects (SOLAR. https://solar-eclipse-genetics.org/about-brief-overview). Each liver-related phenotype (AST, ALT, AST/ALT, CAP, FAST, and kPa), as well as hemoglobin A1C (HgA1C), BMI, and the ARSMA-II Marginalization Scale, was regressed against age, sex, age-squared, sex-by-age, and sex-by-age-squared. The regression residuals derived for each trait were then normalized using an inverse normal transformation [50].

### 2.2. Genotype-by Environment (G×E) Interaction Model for Continuous Environments

The process is a multi-step analysis that begins with a standard additive polygenic model used to obtain estimates of liver trait heritabilities and that serves as a reference model for more complex model development. The polygenic model postulates that the phenotypic covariance can be decomposed into additive genetic and residual environmental variance components. The inter-individual covariances are defined by the additive genetic variance weighted by the genetic relatedness coefficient, assuming (for genetic covariance) that the pairwise genetic correlation across environments is unity and that the additive genetic variance is homogeneous.

The second step is to fit a G×E model to the data. To capture any potential interaction between the genetic effects (either the additive genetic variance and/or genetic correlation) and the specific environment (marginalization), we express both the additive genetic variance and genetic correlation as continuous functions of the environment (marginalization). If the expression of the aggregate of all genotypes underlying a phenotype (polygenotype) is dependent on a specific environment, this implies that the genotype–phenotype map for the trait (NAFLD) is a function of the environment. The G×E interaction variance is zero if the following two conditions are simultaneously true: (1) homogeneity of the additive genetic variance across environments: σ^2^_g1_ = σ^2^_g2_ = σ^2^_g_, where σ^2^_g1_ and σ^2^_g2_ are the additive genetic variance in environments 1 and 2, respectively; (2) complete positive pleiotropy (i.e., the same genes are active across environments) in which the genetic correlation (ρ_g_ = 1) is one across environments: ρ_g_ = 1. There is G×E evidence if either null hypothesis is rejected [51]. Rejection of either or both of these statistical null hypotheses is evidence that the phenotypic response to the environment has a genetic basis.

We modeled the genetic variance and cross-environment genetic correlation as functions of “marginalization”, where the quantitative measure of marginalization is the total score on the marginalization survey. Since it is likely that our focal “environment” is also influenced (directly or, more likely, indirectly) by genetic factors, we first tested for genetic factors underlying the ARSMA-II measure of marginalization and observed a significant heritability of 0.30 (*p* < 3.8 × 10^−5^). We can account for additive genetic and environmental covariances among relatives in a known pedigree structure—using best linear unbiased prediction (BLUP) [52]. We then subtract the BLUP genetic values for the marginalization variable to obtain a BLUP-computed marginalization variable that reflects primarily environmental effects [52]. This lattermost variable is the focal (genetically corrected) environment in our G×E model.

We then model the genetic variance function (and model the environmental variance) using an exponential function of marginalization, where the exponential function maintains positivity, which is required of a variance [53]. Taking the natural logarithm of the exponential function, the variance homogeneity null hypothesis holds for a slope term equal to 0: γ_g_ = 0. The genetic correlation was modeled using the exponential decay function of the pairwise differences in marginalization scores.

We carried out model evaluations and hypothesis testing in two stages. In stage one, we determine which model (overall G×E interaction model or the polygenic model) provided a better fit to the data by way of a likelihood ratio test (LRT). It is important to note that the polygenic model is fully nested within the G×E interaction model and that relative to the polygenic model, the G×E interaction model has three additional parameters (γ_g_, γ_e_, and λ; α_g_ and α_e_ are re-parameterized versions of the variances). The LRT statistic for this comparison is distributed as a 50:50 mixture of chi-squares with 2 and 3 degrees of freedom (df) [50,51,54].

We examine the more specific G×E interaction hypotheses in the second stage. The full G×E model with all parameters estimated was compared with models when either gamma (γ) or lambda (λ) was constrained to 0 to test, respectively, the hypotheses of additive genetic variance homogeneity and a genetic correlation equal to one. The distributions of the LRT statistics are a chi-square with 1 df, and a 50:50 mixture of a chi-square with a point mass at 0 and a chi-square with 1 df [51,54]. As part of this stage, we determined if each of the three additional parameters in the full G×E interaction model (γ_g_, γ_e_, and λ) should even be included at all by comparing its maximum likelihood estimate (MLE) to its standard error (S.E.). A parameter is roughly significant if its MLE is greater than twice its S.E. based on likelihood theory. Therefore, if a parameter S.E. was greater than its MLE, we judged that parameter to be statistically unimportant. We formally tested additional parameters by the same tests. If any of the three additional parameters were found to have S.E.s greater than their MLEs and if these were found to be formally insignificant, we then compared a reduced version of the G×E interaction model to the polygenic model, excluding the insignificant parameters.

## 3. Results

The characteristics (age, HbA1c, marginalization, AST, ALT, AST/AST, BDI-II, BMI, CAP, FAST, kPa) of the sample by sex are listed in Table 1. Two-sample independent *t*-tests tested sex differences. There were significant differences by sex for AST, ALT, AST/ALT, BDI-II, and FAST. There were no significant differences between males and females for HbA1C, marginalization, BMI, CAP, and kPa. 

*Heritability:* Individuals with a complete data set (547) were analyzed. Heritabilities of the traits are significant and reported in Table 2 HbA1c (h^2^ = 0.52, *p* = 2.5 × 10^−6^), Marginalization (h^2^ = 0.30, *p* = 3.8 × 10^−5^), AST (h^2^ = 0.25, *p* = 0.029), ALT (h^2^ = 0.41, *p* = 6.9 × 10^−3^), AST/AST (h^2^ = 0.2 7, *p* = 1.9 × 10^−3^), BDI-II (h^2^ = 0.36, *p* = 1.5 × 10^−5^), BMI (h^2^ = 0.55, *p* = 8.0 × 10^−7^), Steatosis CAP (h^2^ = 0.34, *p* = 3.6 × 10^−4^), FAST (h^2^ = 0.35, *p* = 2.3 × 10^−3^), hepatic fibrosis kPa (h^2^ = 0.37, *p* = 0.01) and steatosis (h^2^ = 0.33, *p* = 4.3 × 10^−3^). We formally compared the full G×E interaction model to the polygenic model for HbA1c, AST, ALT, AST/ALT, BDI-II, BMI, CAP, kPa, FAST (Table 3). 

From likelihood theory, a parameter’s maximum likelihood estimates (MLEs) should be at least two times its standard error (S.E.) to indicate significance. When we found that the S.E. of a parameter was larger than its MLE, and if formal testing revealed it to be insignificant, we then elected to use a reduced version of the G×E model (Red. G×E). The results of comparing the polygenic model to either a reduced or full version of the G×E model are reported in Table 3. We found that the G×E model fit the data significantly better than the polygenic model for A1C, AST/ALT, BDI-II, BMI, and CAP.

**Figure 1:**
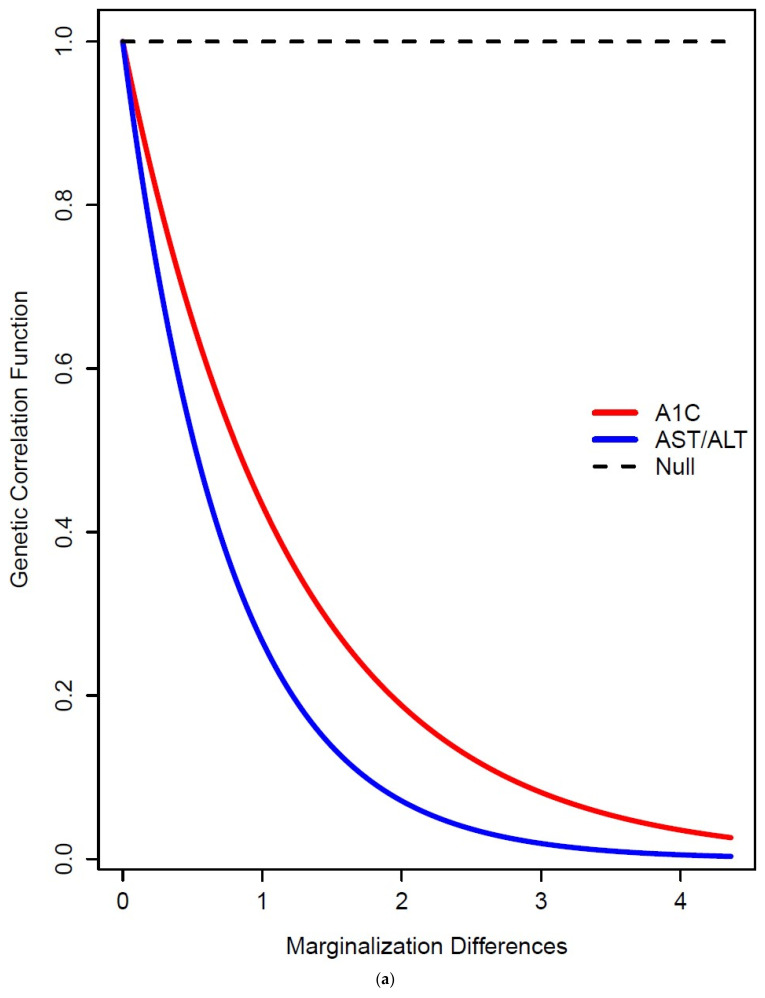

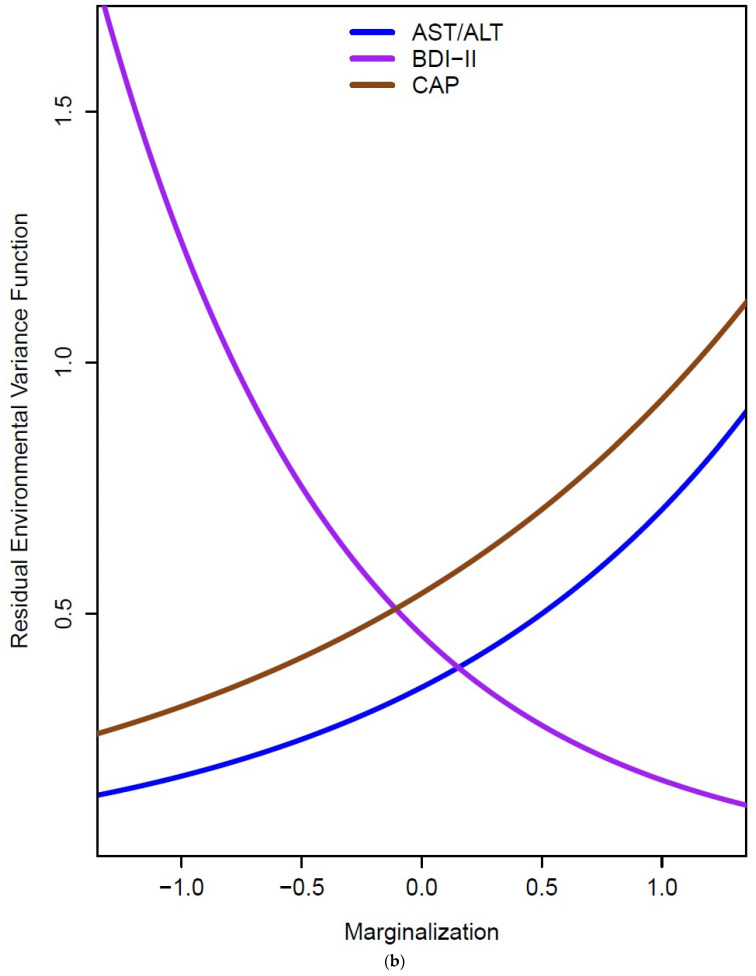

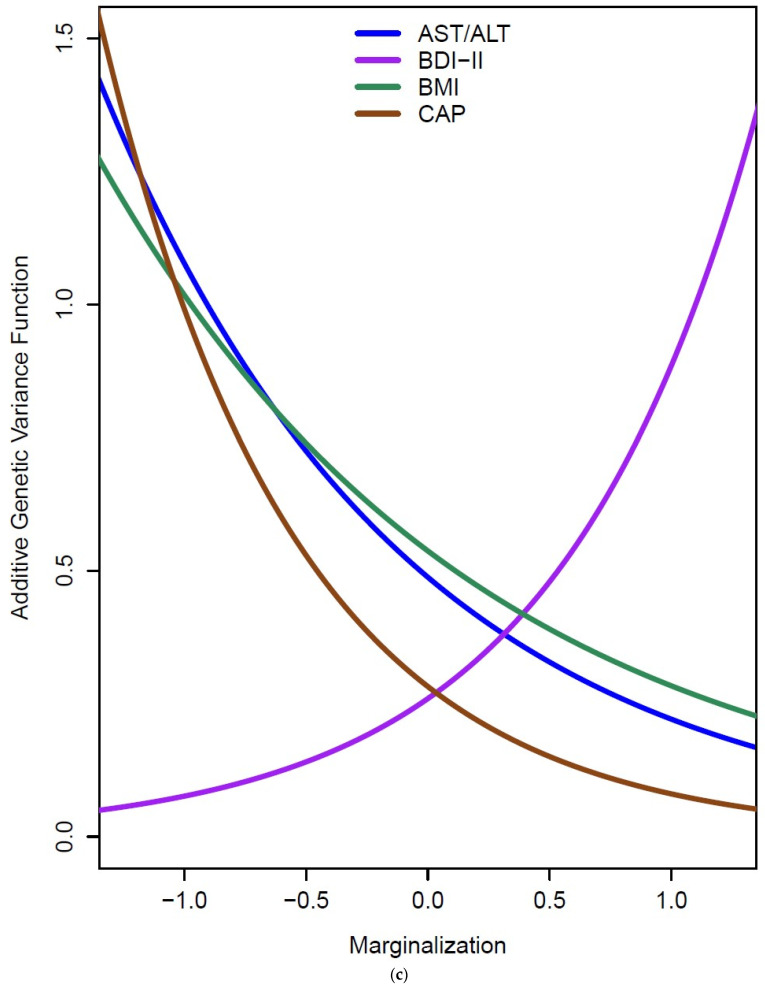
(**a**) The additive genetic variance function AST/ALT, BDI-II, BMI, CAP; (**b**) the residual environmental variance function for AST/ALT, BDI-II, CAP; (**c**) the genetic correlation function for A1C and AST/ALT.

Results of the hypothesis tests for variance homogeneity (additive genetic and/or residual environment) and for the genetic correlation are presented in Figure 1a–c (Addendum Table 1). For HbA1ccc, we found that the genetic correlation significantly decays from one with increasing differences in marginalization (that is, G×E interaction due to the genetic correlation is indicated). For AST/ALT ratio, we found evidence of G×E interaction due to both additive genetic variance heterogeneity and a genetic correlation less than one. Moreover, for this trait, there was significant residual environmental variance heterogeneity. For BDI-II, the additive genetic variance was significantly increasing, whereas the residual environmental variance was significantly decreasing, with increasing marginalization. In other words, BDI-II comes to be increasingly dominated by genetic effects with increasing marginalization. For BMI, we found evidence of G×E interaction due to additive genetic variance heterogeneity (decreasing with increasing marginalization). For CAP, we found significant heterogeneity for both the additive genetic variance (evidence of G×E interaction) and the residual environmental variance. This evidence confirms that genetic factors interact with marginalization to influence the expression of the AST/ALT ratio, as well as risk for diabetes (HbA1c), depression, and obesity (BMI).

## 4. Discussion

This study investigates the gene-by-environment interactions and the response to acculturation stress (marginalization) that shape chronic disease (obesity, diabetes, depression, and liver disease) risk. The impact of an environmental factor (such as marginalization) on a trait (such as obesity, diabetes, depression, and liver disease) can vary depending on the genetic makeup of an individual. G×E interaction findings increasingly support how genes underlying chronic illness respond to different environments. Our recent studies demonstrate the joint effects of socioeconomic status and genetics on risk for cardiovascular disease [25]. We used variance component models and likelihood-based statistical inference to examine the role of genes underlying hepatic fibrosis, steatosis, transaminases, and the FAST score that respond to the environment (depression). We found statistically significant moderate heritabilities for AST/ALT (h^2^ = 0.26, *p* = 0.004), (3) FAST (h^2^ = 0.36, *p* = 1.5 × 10^−3^) [3], fibrosis (h^2^ = 0.36, *p* = 0.01), and steatosis (h^2^ = 0.33, *p* = 0.01) [16]. We also reported G×E interaction between genes underlying the AST/ALT ratio, FAST, steatosis, fibrosis, and depression [3,16]. In this study, we found associations between marginalization, obesity, diabetes, depression, and NAFLD (in particular, the AST/ALT ratio and steatosis), further affirming the G×E effects at the molecular level. 

Acculturation affects lifestyle habits among Hispanics in United States–Mexico border communities [50]. Less acculturated Mexican Americans are more likely to consume food items on the Healthy Eating Index [51]. Second-generation Mexican Americans who prefer English, compared to bilingual or Spanish language preference (especially women), more frequently report eating foods on the Unhealthy Eating Index. Mexican Americans born in Mexico report consuming more fruits and vegetables than Mexican Americans born in the US. Recent immigrants report healthier eating habits and fewer sugary drinks than second- and third-generation Mexican Americans. When controlled for sociodemographic factors, health, dietary intake, physical activity, and sedentary behavior, greater acculturation was associated with higher BMI and unhealthy dietary intake. 

Older individuals demonstrate a greater likelihood of consuming foods on the Healthy Eating Index and of not consuming foods on the Unhealthy Eating Index. Acculturation and hypertension in Mexican Americans are associated (aOR = 1.71, 95% CI, 1.24–2.36) [52]. Our findings reproduce other research as the effects of a genetic risk score for increased BMI and female sex are worsened with higher levels of acculturation in Mexican Americans (controlled for relatedness, gender, principal components of ancestry, and gender) [53]. Acculturation is an effect modifier of family history on metabolic syndrome, correlating with the number of years living in the US. As the number of years in the United States increased, the effect of family history on metabolic syndrome increased, suggesting that gene-by-environment interaction may be more critical in metabolic syndrome traits compared to type 2 diabetes mellitus (T2DM) [54]. The length of residency in the United States is also one of the strongest predictors of obesity. A total of 16% of the variation in BMI can be attributed to the age of immigration, years since the first arrival to the US., and healthy diet [53]. For obesity, the environment may be more important than genetic factors [55].

The process of acculturation that mediates adverse physical and mental health outcomes [56] is explained by acculturation stress experienced from negotiating heritage and host cultures. Age, female sex, acculturation stress, and emotional support are related to chronic illness, while ethnic pride, coping, social support, and individual resilience buffer adverse health outcomes [57]. Our findings support significant genetic variation for BMI and diabetes in response to acculturation. 

NAFLD is highly prevalent in both American-born and Mexican-born individuals. Individuals of Mexican ancestry are carriers of risk alleles for metabolic syndrome, diabetes, and depression [28,58,59,60]. Immigration status, length of stay in the United States, socioeconomic status, education, and chronic illness affect the prevalence of NAFLD and progression to fibrosis and hepatocellular carcinoma. Perceived stress, differences in experiences, and personal resilience mediate the stress response and are also NAFLD risk factors [61]. The odds of NAFLD are higher for Mexican-origin adults with an Anglo orientation, and the odds are lower for Mexican-origin individuals with a Mexican orientation, both reflecting the role of stress associated with acculturation. 

Patients with NAFLD have a higher prevalence of depression than patients without NAFLD (R.R.: 2.83, *p* < 0.001) [14,62], and patients with depression have a higher prevalence of NAFLD [3,16]. Our current findings support the G×E interaction between NAFLD, depression, and acculturation and support the role of acculturation in the phenotypic expression of other chronic illnesses (obesity and diabetes) [63]. NAFLD is a complex condition influenced by genetic, environmental, and social factors. The immune inflammatory response is to be a mediator of chronic illnesses such as diabetes, obesity, depression, and liver disease. Our findings suggest that the interplay of NAFLD and acculturation stress (marginalization) occurs at the cellular or molecular level [57,64,65,66,67,68]. Stress may activate behavioral, psychological, and physical reactions. Pro-inflammatory pathways are involved in translating sociocultural stress (socioeconomic status and low education levels) into mental and physical health disparities. There is evidence that many chronic illnesses upregulate pro-inflammatory gene expression. The reactions are mediated by the hypothalamus–pituitary–adrenal axis (corticotropin-releasing hormone, adrenocorticotropic hormone, and glucocorticoids) and the sympathetic–adrenal–medullary axis. The pathways are mediated by the immune response (chemokines, cytokines, and growth factors), including interleukin 6 (IL6), interferons (IFNs), and tumor necrosis factors (TNFGs) [69]. Job stress has been associated with seven serum factors (CCL27 CCL27/CTACK, CCL11/Eotaxin, CCL2/MCP-1, IL-17, IL-6, and BDNF) [69]. While the PNPLA3 polymorphism rs738409 C/G variant has been reliably associated with an increased prevalence of NAFLD and the progression of the disease to NASH and hepatocellular carcinoma [70], little is currently known about specific genes involved in NAFLD risk. Future gene identification and transcriptomic, proteomic, and network analysis will further highlight possible precision medicine approaches to the management of chronic illnesses. 

## 5. Limitations

The cross-sectional nature of this study limits our ability to infer causality between acculturation stress, gene-by-environment interactions, and the prevalence of chronic illnesses such as NAFLD, obesity, diabetes, and depression. Longitudinal studies are needed to confirm these associations over time. While our study provides valuable insights into the gene–environment interaction between NAFLD and marginalization, it is essential to acknowledge certain limitations when interpreting the findings. The Acculturation Rating Scale for Mexican Americans-II (ARSMA-II) provides valuable insights but may not capture the full complexity of the acculturation process. Other dimensions of acculturation, such as cultural practices, social networks, and identity conflict, may also influence health outcomes and should be explored in future research. While we controlled for several environmental factors, other unmeasured variables, such as specific dietary components, occupational hazards, and detailed socioeconomic factors, could confound the observed relationships. Future studies should aim to include a broader range of environmental and lifestyle variables. Our sample of 547 participants is reasonable for a single-center study but may limit the generalizability of the findings. The population of the Rio Grande Valley region is predominantly of Mexican origin. A more extensive and diverse sample would provide a broader representation of the Mexican American population and more robust results. The study design was cross-sectional (data from a single time point) and limits, as mentioned, our ability to establish causal relationships or determine the directionality of the observed associations. Longitudinal studies can elucidate the temporal dynamics of the gene-by-environment interactions and their impact on NAFLD, depression, obesity, diabetes, and depression. Our focus is on Mexican Americans, and future research will determine whether the finding of genotype-by-environment interaction effects between chronic illness and acculturation is replicated in other populations. Finally, we did not evaluate the role of alcohol in the G×E interaction of NAFLD and marginalization. Alcohol consumption may affect the interaction, but that is also true for many chronic and acute illnesses. Self-reported alcohol intake is not a sensitive measure of alcohol use. However, future studies could include biomarkers such as ethanol, ethyl glucuronide, ethyl sulfate, phosphatidyl ethanol, fatty acid ethyl esters, and acetaldehyde. 

## 6. Conclusions

This study investigates the gene-by-environment interactions that influence the prevalence and progression of chronic illnesses such as obesity, diabetes, depression, and nonalcoholic fatty liver disease (NAFLD) among Mexican Americans in the Rio Grande Valley. The findings highlight the significant role of acculturation stress, particularly marginalization, in exacerbating these conditions. The research underscores the heritability of these traits and the complex interplay between genetic predispositions and environmental factors. While the study provides valuable insights, it also acknowledges limitations such as the need for larger, more diverse sample sizes and longitudinal studies to establish causality. Future research should aim to explore additional dimensions of acculturation and include a broader range of environmental and lifestyle variables to enhance our understanding of these interactions. Addressing these factors is crucial for developing targeted interventions to mitigate the high prevalence of NAFLD and associated comorbidities in this population. Future directions will focus on identifying the nature of the interactions and the specific genes involved.

## Figures and Tables

**Table 1:** Characteristics of the sample showed significant differences by sex for AST, ALT, AST/ALT, BDI-II, and FAST.

Trait	Females		Males		*p*-Value for Difference
	Mean (N = 406)	S.D.	Mean (N = 141)	S.D.	
Age	44.13	14.61	46.18	15.69	0.1739
Marginalization	24.21	10.96	24.43	12.02	0.8543
BDI-II	6.79	8.14	4.59	6.51	1.4 × 10^−3^
BMI	32.49	7.07	32.25	6.97	0.7272
HbA1C	6.60	1.96	6.62	2.13	0.9318
AST	19.91	15.78	25.69	19.83	2.0 × 10^−3^
ALT	22.30	23.73	33.35	31.98	1.2 × 10^−4^
AST/ALT	1.16	0.65	0.99	0.63	6.4 × 10^−3^
CAP	284.24	64.94	289.64	59.21	0.4919
kPa	6.64	4.49	7.02	4.11	0.4808
FAST	0.14	0.20	0.22	0.26	8.4 × 10^−3^

**Table 2:** Significant heritabilities with *p*-values.

Trait	Heritability	Standard Error	*p*-Value
HbA1C	0.52	0.11	2.5 × 10^−6^
Marginalization	0.30	0.08	3.8 × 10^−5^
AST	0.25	0.14	2.0 × 10^−2^
ALT	0.41	0.13	6.9 × 10^−3^
AST/ALT	0.27	0.10	1.9 × 10^−3^
BDI-II	0.36	0.10	1.5 × 10^−5^
BMI	0.55	0.11	8.0 × 10^−7^
CAP	0.34	0.10	3.6 × 10^−4^
FAST	0.35	0.13	2.3 × 10^−3^
kPa	0.33	0.13	4.3 × 10^−3^

**Table 3:** Testing the G × Marginalization interaction model against the polygenic model demonstrating G×E interaction.

Trait	Model	Ln Likelihood	Chi-Square	*p*-Value
HbA1c	Polygenic	−261.838	9.72868	4.8 × 10^−3^
	Reduced G×E	−256.973		
AST	Polygenic	−261.088	2.503294	0.199821
	Full G×E	−259.836		
ALT	Polygenic	−264.444	2.952168	0.157147
	Full G×E	−262.968		
AST/ALT	Polygenic	−265.081	14.65585	3.9 × 10^−4^
	Full G×E	−257.753		
BDI-II	Polygenic	−247.356	20.96177	1.6 × 10^−5^
	Full G×E	−236.876		
BMI	Polygenic	−260.745	12.40074	2.03 × 10^−3^
	Reduced G×E	−254.545		
CAP	Polygenic	−261.838	9.72868	7.7 × 10^−3^
	Reduced G×E	−256.973		
FAST	Polygenic	−226.611	1.992278	0.263703
	Full G×E	−225.615		
kPa	Polygenic	−275.19	0.375754	0.684301
	Full G×E	−275.002		

## Data Availability

The raw data supporting the conclusions of this article will be made available by the authors on request.
